# Vertiginous Symptoms Consecutive to Clipping in the Horse

**Published:** 1890-01

**Authors:** 

**Affiliations:** Veterinarian at Chalons


### VERTIGINOUS SYMPTOMS CONSECUTIVE TO CLIPPING IN THE HORSE.

By Dr. Guibert,
*Veterinarian at Chalons.*

Recueil de Med. Vet., November 1889.

*History:* A bay gelding, seven years old, had been regularly worked in the threshing machine, and fed daily six quarts of oats, two quarts of bran, ten pounds of hay, and about fifteen pounds of wheat straw, had not worked that morning, and at noon had eaten his regular feed, was found at five in the afternoon pushing his head against the wall and moving his legs in a disordinate manner.

*Symptoms:* On my arrival, the horse was standing completely immobile, with the four legs drawn together, the anterior ones were carried back and the posterior slightly under the body, seemed to push it forward. The summit of the head resting against the wall in the left-hand corner of the stall, and the chest against the manger, held the animal and prevented it from falling forward. The tuft of the chin, on account of the bending in of the head, touched the internal face of the front of the manger, but I had scarcely time to observe the animal's position when the horse had an attack. He struck the legs violently, principally the posterior ones, in all directions, pushing strongly against the wall as if he wished to throw himself forward, and after a few minutes of agitation took again the position of immobility, described above. The respiration, interfered with by the bend-

[1] It being desirable to preserve the brain for other purposes.

ing in of the head, was slowed, and accompanied by a peculiar snoring. The conjunctiva, which I examined by the aid of a lamp, after climbing into the manger, presented nothing abnormal, except, perhaps, as to coloring, which was a little more marked than in health. The mouth was moist and soft to the touch, the arteries soft and supple, the pulse full and accelerated (about 70), impossible to take the temperature.

The history and symptoms, which I would call negative, resulting from the examination—superficial, it is true—of the conjunctiva, of the buccal mucus membrane, of the arteries and pulse, the shortness of the attack—one or two minutes at the most—left a doubt in my mind. Instead of my answering my client, who asked me if it was the staggers, I questioned him again; asking him if his horse had suddenly changed his diet, if he had been over-driven, or beaten by one of the attendants, or had he been frightened by any one in the afternoon. He assured me again that the feed had not been changed, no one had beaten or over-driven the animal, as he had driven him himself, and that he could not have received any fright, the stable being completely closed, and no one had been in it during the afternoon. In view of these facts, I resolved to examine the animal outside. I procured a long rope, which was put on the halter, and gave it to three men, who were placed in the neighboring stall on the right, and ordered to pull suddenly and strongly to the right and backward. The animal pivoted, as it were, on himself, and bolted forward, dragging me as well as my assistants, but on arriving in the court yard stopped, with his head, held by my assistants, strongly drawn in and the four legs in a bunch. In a few minutes he lifted and replaced the right hind leg, lifted it again and carried it backward; then displaced the anterior ones in the same way, so that at the end of four or five minutes he had taken a natural position. I ordered my assistants to let go of the head, and immediately the animal looked about to the right and left, snorted loudly and seemed astonished to find himself free. The respiration and pulse became normal, the horse could be backed and moved in all directions without difficulty. Surprised by this change, and not knowing to what to attribute the symptoms of vertigo, I returned the horse to the stall. He was scarcely in, when he struck with his legs in all directions, threw himself forward to the left, pushed against the wall and took again the position of immobility, before described. Continuing to watch my patient, I noticed that the attacks came on each time that, fatigued by being in an awkward position, he attempted to replace a leg. I took him outside again to examine the extremities, and as in the first case, after a few moments of hesitation, the horse took a natural position of his own account. I passed my hand over the legs without noticing anything; however, having seen that the hairs had been recently clipped, I questioned my client on the subject, and he informed me that he had only been clipped that morning. Passing the hand again with more care over the hind legs, I noticed a sort of twitching and nervousness, when I touched the pasterns lightly. Suspecting the cause of the trouble, I took some whisps of straw and touched the posterior fetlocks lightly. The horse with a fright began snorting loudly, and bolted forward dragging the men who held him. The second and third trial gave me the same result. In order to assure myself that this irritability, these symptoms of vertigo were really produced by the tickling of the straw, and the result of an erethism of the hair bulbs following the clipping, I removed all the bedding from the stable and returned the horse to it. All drawn up, he appeared at first uneasy, looked about him, and then after some hesitation he backed and started to eat his oats which I had put in the manger. From this moment the cause having been removed, the attacks ceased. Since this, I have noticed several times that I could provoke in this horse, ordinarily of a quiet temperament, brusque movements of the legs, and nervous irritation, characterized by elevation of the head, rapid movements of the ears, front and back, dilation of the nostrils, and a startled look of the eye, by touching lightly with a little stick the pastern and the coronary band.

Is there an analogy between this sensibility and that so well known of the planter surface of the foot in man? I do not know, and will not discuss the question, but report this case which I believe is rare, to warn the young practitioner against a possible error of diagnosis.

